# SmellyCode++: Multi-Label Dataset for Code Smell Detection

**DOI:** 10.1038/s41597-025-05465-z

**Published:** 2025-07-12

**Authors:** Nawaf Alomari, Amal Alazba, Hamoud Aljamaan, Mohammad Alshayeb

**Affiliations:** 1 Information and Computer Science Department, King Fahd University of Petroleum and Minerals, Dhahran, Saudi Arabia; 2 Department of Information Systems, King Saud University, Riyadh, 11362 Saudi Arabia; 3 Interdisciplinary Research Center for Finance and Digital Economy, King Fahd University of Petroleum and Minerals, Dhahran, Saudi Arabia; 4 Interdisciplinary Research Center for Intelligent Secure Systems, King Fahd University of Petroleum and Minerals, Dhahran, Saudi Arabia

**Keywords:** Scientific community, Mathematics and computing

## Abstract

**Context:** Code smells indicate poor software design, affecting maintainability. Accurate detection is vital for refactoring and quality improvement. However, existing datasets often frame detection as single-label classification, limiting realism. **Objective:** This paper develops a multi-label dataset for code smell detection, integrating textual features and numerical metrics from open-source Java projects. **Method:** We collected code from 103 Java projects, parsed it into Abstract Syntax Trees (ASTs), extracted features, and annotated samples based on prior studies. Data cleaning, unification, and merging techniques were applied to support four code smells: God Class, Data Class, Feature Envy, and Long Method. **Results:** The dataset comprises 107,554 samples with multi-label annotations, improving detection realism. Evaluation shows F1 scores of 95.89% (Data Class), 94.48% (God Class), 88.68% (Feature Envy), and 88.87% (Long Method). **Conclusion:** This dataset aids advanced studies on code smell detection, particularly for fine-tuning LLMs. Future work can expand it to other languages and additional smells, enhancing diversity and applicability.

## Background & Summary

Code smells are not software defects but rather indicators of suboptimal design choices that can adversely impact software quality attributes, such as maintainability, over time^1,2^. These smells can appear at various levels of the codebase, including class-level issues, such as the “God Class” and “Data Class,”^3^ as well as method-level issues, such as the “Long Method” and “Inline Method”^3^.

Modern software systems play a critical role in our daily lives and are becoming increasingly complex. Consequently, maintaining such systems exceeds their initial development cost^4^. It is essential to detect and address code smells to mitigate maintenance costs and preserve codebase quality. Detecting code smells enhances software quality and prevents long-term technical debt^5^. A recent systematic mapping study covering 18 years of research demonstrates the breadth of detection approaches, terminology, and validation practices that have emerged in the domain^6^.

Refactoring techniques offer a solution to mitigate code smells by restructuring code without altering its external behavior^3^. Alshayeb^7^ examined the impact of refactoring on software quality attributes by comparing metrics before and after applying specific techniques, but found inconsistent results. Later, Elish and Alshayeb^8^ provided a categorization of refactoring techniques based on their impact on quality attributes, facilitating the selection of refactoring methods aimed at enhancing specific software properties. However, detecting code smells is a prerequisite for effective refactoring.

Recent advancements in fine-tuning language models have shown success across tasks like text classification^9,10^, question answering^11,12^, and more. There has also been growing interest in applying fine-tuning and machine-learning techniques for code smell detection^13–16^. Emerging work has even explored privacy-preserving, federated settings—enabling multiple organizations to collaboratively train smell detectors without sharing raw code^17^. Comparative evaluations further reveal that a wide range of mainstream classifiers achieve consistently high accuracy for God Class detection, even when the training data remain highly imbalanced^18^. Empirical evidence also indicates that enriching training data with project-specific context information (e.g., domain and size) significantly boosts detection performance^19^.

Despite promising results, fine-tuning models for code smell detection requires extensive, high-quality datasets. Several efforts have been made to construct code smell datasets^20–24^; however, most of these datasets are designed for single-label classification^14,20– 25^. Only a few studies have developed multi-label code smell datasets^15,26,27^. However, these datasets often contain a limited number of instances or are collected from a small number of projects, which restricts their generalizability. Complementary investigations suggest that incorporating high-level project status information can further improve Large Class detection without extensive feature engineering, underscoring the value of richer contextual features in new datasets^28^.

The nature of code smells is more suited to multi-label classification, as a single code sample can exhibit multiple smells simultaneously. For instance, a method could demonstrate both the “Feature Envy” and “Long Method” code smells. Constructing a multi-label code smell dataset can improve detection accuracy by enabling models to learn more realistic patterns encountered in real-world software projects.

Moreover, most existing studies have focused on extracting code metrics as features for the detection process, a technique widely adopted in previous research^14,15,25,27^. However, relying solely on extracted metrics can limit the dataset’s applicability for fine-tuning code language models for code smell detection tasks. Additionally, information loss may occur, as the metrics represent only a subset of the characteristics of the actual code samples.

To address these limitations, our contribution lies in constructing a multi-label code smell dataset that includes both textual code samples and numerical code metrics. This approach supports fine-tuning language models and traditional machine learning techniques, enhancing the dataset’s adaptability and potential impact.

The main objective of this work is to construct a code smell dataset for the purpose of multi-label classification tasks with respect to textual features and numerical code metrics from the point of view of researchers in the context of Java open-source projects.

In this study, we focus on four prominent code smells. These specific code smells were selected due to their significant negative impact on software quality^6^. The chosen code smells, along with their descriptions, are as follows:

- **God Class**^29^: Sometimes called a Large Class^3^, it is a class that takes on too many responsibilities and should be split into smaller, more focused classes.
- **Data Class**^3^: A class that contains only data attributes without any associated methods.
- **Long Method**^3^: A method that is excessively long or manages extensive logic, which could be divided into smaller, more manageable methods.
- **Feature Envy**^3^: A method that frequently accesses data fields of another object more often than its own, indicating it may belong to a different class.

## Related Work

This section reviews the literature on constructed datasets for code smell detection in Java. We categorize datasets by the labeling approach into two clusters: single-label and multi-label. In the single-label approach, each sample has exactly one label, whereas in the multi-label approach, a sample can be associated with more than one smell label.

### Single Label

Most of the reviewed studies have adopted a single-label approach, and these studies can be summarized in Table 1. In this approach, each code sample in the dataset is annotated with only one code smell as a class label^22,24,25^. Alternatively, some datasets are divided for binary-classification purposes, where each subset contains samples that either exhibit a specific smell or not^14,20,21^. While the single-label approach is widely used, it has limitations in real-world scenarios where a single code sample may contain multiple smells simultaneously. This restricts the ability of machine-learning models to capture complex patterns involving multiple smells.

**Table 1:** Summary of related work for Single Labels.

Ref	Smells	Proj.	size	Feature	Smells covered
Palomba *et al*.^23^	5	30	243	Textual	Blob, parallel inheritance, shotgun surgery, divergent change, feature envy
Arcelli Fontana *et al*.^20^	4	74	1 680	Numeric	God class, data class, feature envy, long method
Fontana and Zanoni^21^	4	76	1 680	Numeric	God class, data class, feature envy, long method
Di Nucci *et al*.^14^	4	74	3 350	Numeric	God class, data class, feature envy, long method
Palomba *et al*.^24^	14	30	40 000	Textual	Class data should be private, complex class, spaghetti code, feature envy, God class, middle man, inappropriate intimacy, parameter list, refused bequest, lazy class, long method, long message chains, speculative generality
Lenarduzzi *et al*.^25^	23	30	37 553	Numeric	Duplicated code, blob, class data should be private, cyclomatic complexity, down-casting, excessive use of literals, functional decomposition, feature envy, God class, inappropriate intimacy, excessively short identifiers, large class, lazy class/freeloader, orphan variable or constant, Swiss army knife, refused bequest, spaghetti code, speculative generality, excessive return of data, tradition breaker, excessively long identifiers, long method, too many parameters / long parameter list

The features examined in the reviewed studies are categorized into two distinct clusters: *numerical* and *textual*. In the numerical cluster, datasets employ extracted code metrics as features, whereas in the textual cluster, datasets use actual code snippets as textual representations.

Several studies have developed datasets for detecting code smells in Java using numerical features—that is, code metrics—as input^14,20,21,25^. These metrics were derived with either custom-built tools^20,21^ or existing tools such as SonarQube^25^.

Arcelli *et al*.^20^ developed a dataset for detecting four specific code smells: God Class, Data Class, Feature Envy, and Long Method. The dataset consists of four distinct files (420 samples each) and includes class- and method-level metrics. The smells were extracted with dedicated tools, and the code came from 74 Qualitas Corpus projects.

Building on this work, Fontana *et al*.^21^ created a dataset that adds a severity level. Severity—on an ordinal scale from 0 to 3—was assigned with their *Advisors* (tool- and rule-based detectors) according to how many times a given smell was identified for each sample.

Similarly, Di Nucci *et al*.^14^ merged several existing resources into four sub-datasets (840 samples each) covering the same four smells. Metrics were extracted at both class and method granularity. The authors relied on the original validations rather than performing a new one.

Lenarduzzi *et al*.^25^ provided a large-scale dataset (almost 37000 instances) with 23 smells and severity levels. They used SonarQube for metrics and Ptidej for smell detection. Despite its size, the dataset lacks independent validation.

Other studies adopted textual features—that is, the raw source code itself^22–24,26^. Such datasets are well suited to NLP pipelines (e.g., embedding extraction or transformer fine-tuning).

Madeyski and Lewowski^22^ released 18 000 textual instances covering Blob, Data Class, Feature Envy, and Long Method. Expert manual labelling and validation ensure reliability.

Palomba *et al*.^23^ built a 243-instance dataset with Divergent Change, Shotgun Surgery, Parallel Inheritance, Blob, and Feature Envy. Two authors labelled the data manually; the small size and inclusion solely of smelly classes limit its usefulness.

Palomba *et al*.^24^ later introduced a 40 000-sample dataset spanning 13 smells across 30 projects of various sizes. Only smelly classes are included; smells were detected with multiple tools and then expert-validated.

### Multi Label

Only a few studies focused on *multi-label* datasets; these studies can be shown in Table 2. In this approach, each sample is annotated with multiple smells simultaneously^15,26^. This approach enables models to learn richer and more realistic patterns.

**Table 2:** Summary of related work for Multi Labels.

Ref	Smells	Proj.	size	Feature	Smells covered
Khomh *et al*.^26^	4	4	194 189	Textual	Anti-singleton, blob, class data should be private, complex class, large class, lazy class, long method, long parameter list, message chains, refused parent bequest, speculative generality, Swiss army knife
Madeyski and Lewowski^22^	4	523	18 030	Textual	Blob, data class, feature envy, long method
Guggulothu and Moiz^15^	2	74	445	Numeric	Feature envy, long method
Hadj-Kacem and Bouassida^27^	8	30	26 932	Numeric	Class data should be private, spaghetti code, complex class, large class, message chain, middle man, lazy class, speculative generality
*This work*	4	103	107 554	Both	God class, data class, feature envy, long method

**Table 3:** Selection criteria for open-source projects.

Criteria	Description	Rational
Industry Diversity	Select different projects from different domains, industries, and districts.	Code from different domains can have different terms and logic.
Project Size	The projects were grouped into clusters based on their size, measured in LOC. While several categorizations for project sizes exist, such as the one proposed by Tempero *et al*.^39^, we adopted an alternative classification scheme that divides projects into five categories: small, medium, semi-large, large, and very large^40^. A diverse selection of projects was then sampled from each of these clusters.	Different project sizes and relationships between packages and classes can have different complexity levels.
Project Maturity	The project maturity can give insights into how old and complex the project is. We used the length of the commit history to measure maturity.	Mature projects can be more complex and have more real-life examples.
Open Source	The project should be open-source	to make it available to everyone and make it possible to replicate.
Availability of Labels	A project that was studied and labeled for the code smell detection task.	As we will use others’ studies’ annotations.

Khomh *et al*.^26^ developed a dataset based on the textual features of the code, supporting 12 smells. However, it was derived from only four open-source projects, which limits its generalizability.

The dataset by Guggulothu and Moiz^15^, created by merging existing resources, is comparatively small (445 samples) and covers only two smells.

Hadj and Bouassida^27^ constructed a multi-label dataset from 30 open-source projects. It relies on code-metric features and includes eight distinct smells at the class level.

### Gap Analysis

This study addresses critical gaps identified in the existing literature. First, there is a clear need for a large multi-label dataset, as current datasets are limited in size or drawn from a small number of projects, reducing their generalizability across different domains. Expanding the availability of multi-label datasets will enable models to capture the complexity of real-world code, allowing for the detection of multiple code smells in a single instance, which better reflects practical scenarios.

Additionally, many studies that provide text-based datasets only include the file path to the class within the open-source project, making it challenging to obtain the actual code, especially across different versions of the project. This limitation hinders reproducibility and accessibility for further research. To address this, we emphasize the need for datasets that include the actual code to streamline access for researchers.

Finally, there is a lack of datasets that integrate both numerical code metrics and the actual code as textual features. Creating a dataset that includes both will be invaluable for comparing the effectiveness of these two approaches, fostering new insights and methodologies for code smell detection, and improving model performance.

## Methods

**Fig. 1:**
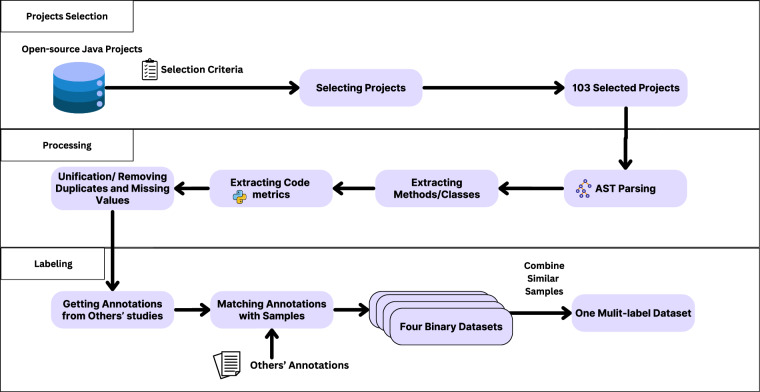
The research methodology.

To construct the dataset, we followed the approach illustrated in Fig. 1. Each step is described in detail below.

### Open Source Project Collection

We began by collecting open-source Java projects from various repositories. To ensure a representative selection, we defined criteria that guided our project choices. The details of these criteria are presented in Table 3. After the projects were selected, they were cloned for further processing. The selected projects are clustered in tables based on the category for better readability and can be shown in Tables 4–9

**Table 4:** Projects in the *Data & Storage* cluster (*n* = 42).

Project	Ver.	NOC	NOM	LOC	Domain
accumulo	1.7.3	3254	33810	419546	Database
apache-atlas-sources	0.8.2	923	8429	108950	Data Governance
apache-calcite	1.21.0	2762	23918	321172	Database Framework
apache-cassandra	0.7.4	636	6876	88612	Distributed DBMS
apache-crunch	0.9.0	568	3384	35261	Data Processing
apache-drill	1.11.0	2942	31495	334522	Interactive SQL
apache-flink	1.4.0	5061	62986	801177	Stream Processing
apache-giraph	1.3.0	318	2136	27693	Graph Processing
apache-gora	0.8	232	1759	20928	In-Memory Data
apache-hadoop	2.6.5	9462	81221	1256230	Distributed FS
apache-hbase	2.0.0	3642	46985	595086	Wide-Column Store
apache-hive	2.3.3	5206	38105	564540	Data Warehouse
apache-hudi	0.7.0	1473	11833	137453	Lakehouse Storage
apache-hyracks	0.3.5	635	5061	629986	Data Flow Engine
apache-iceberg	0.11.0	1088	7266	98928	Table Format
apache-ignite	2.8.0	4786	48945	743648	In-Memory DB
apache-impala	3.2.0	2620	18094	321437	MPP SQL Engine
apache-kudu	1.12.0	1863	14089	238029	Columnar Store
apache-lens	2.7.1	531	3755	62101	BI Framework
apache-mahout	0.14.0	900	5661	87246	ML Library
apache-marmotta	3.4.0	504	3408	60191	Linked-Data Store
apache-nifi	1.11.4	7265	53671	704566	Data Flow
apache-oozie	4.3.0	400	4301	63095	Workflow Scheduler
apache-orc	1.5.6	650	4763	69928	Columnar Format
apache-parquet-mr	1.11.1	637	4936	68220	Columnar Format
apache-phoenix	4.14.0	2392	17543	242812	SQL Layer
apache-pinot	0.7.0	1654	10828	166050	OLAP Store
apache-ranger	2.0.0	1211	10801	132142	Security Service
apache-samoa	0.5.0	176	1396	19073	Streaming ML
apache-shardingsphere	5.0.0	4895	34026	459159	DB Sharding
apache-solr	7.5.0	2212	20735	319872	Search Platform
apache-spark	2.4.8	6712	50547	982972	Analytics Engine
apache-storm	1.2.3	1475	15240	174014	Stream Processing
apache-tajo	0.13.0	1258	10432	150312	Relational Query
apache-tephra	0.15.0	285	1777	24238	Transaction Engine
apache-tika	1.19	584	4618	63841	Content Analysis
apache-zeppelin	0.9.0	620	6397	77699	Data Analytics UI
druid	0.14.2	2625	19901	297862	OLAP Store
kudu-tserver	1.12.0	128	987	10856	Columnar Store
presto	330	4728	33927	548215	SQL Query Engine
pulsar-broker	2.5.0	2118	20641	287382	Message Streaming
rocksdb	6.6.4	1854	14483	177137	Embedded DB
spark-sql	2.4.8	677	8381	122650	SQL Module

**Table 5:** Projects in th e *DevOps & Build Tools* cluster (*n* = 32).

Project	Ver.	NOC	NOM	LOC	Domain
apache-ant	1.7.0	1267	11282	117076	Build Automation
apache-archiva	1.4.M3	760	6955	92045	Artifact Repo
apache-arrow	0.14.1	1809	13830	202054	In-Memory Col Fmt
apache-avro	1.9.1	786	6270	86158	Data Serialization
apache-beam	2.19.0	2124	14483	185612	Unified Batch/Stream
apache-buildr	1.5.0	275	1641	25936	Build Tool
apache-cmake	3.15.2	641	4060	59123	Build System
apache-ducc	2.2.0	604	4617	83902	Cluster Mgmt
apache-gradle	5.6.4	2650	18765	267823	Build Automation
apache-groovy	2.5.8	2017	17940	274253	Language/Build
apache-guacamole	1.1.0	261	2905	53772	Remote Desktop
apache-jmeter	5.2.1	1038	7970	99483	Load Testing
apache-just	0.4.0	179	1423	22531	Build Tool
apache-maven	3.6.3	308	2959	43101	Build Automation
apache-maven-plugins	3.1	2488	14333	187251	Build Plugins
apache-netbeans	11.2	6077	35602	603341	IDE
apache-nexus	3.21.1	1401	11862	196708	Artifact Repo
apache-sbt	1.3.5	1933	16327	251412	Build Tool (Scala)
apache-surefire	3.0.0	565	3792	56676	Test Runner
apache-thrift	0.13.0	2442	15177	227650	RPC Framework
bazel	2.0.0	3415	28451	438902	Build System
buck	2020.02.04	2034	14410	257341	Build System
gradle	6.2.2	3912	28117	411945	Build Automation
guice	4.2.3	246	1901	27721	DI Framework
ivy	2.5.0	701	5806	69024	Dependency Mgmt
jenkins	2.235.1	4067	33120	558502	CI Server
mrjob	0.7.4	189	1399	18264	MapReduce Jobs
packer	1.5.5	1855	15120	237950	Image Builder
sbt-plugins	1.0	927	6480	97235	Build Plugins
teamcity	2019.2	2601	20987	357821	CI Server
vagrant	2.2.7	2018	17195	249390	Dev Env Manager
velocity	2.2	672	5457	68602	Template Engine
xerces2-j	2.12.0	883	9799	142696	XML Processing

**Table 6:** Projects in the *Integration & Messaging* cluster (*n* = 9).

Project	Ver.	NOC	NOM	LOC	Domain
activemq-parent	5.15.7	3534	28867	295784	Message Broker
apache-artemis	2.21.0	4255	39407	484235	Message Broker
apache-camel	3.11.0	2009	10507	99145	Integration Framework
apache-kafka	1.0.0	2877	17611	245364	Messaging System
apache-kafka-connect	1.0.1	532	4070	52802	Stream Processing
camel-spring-boot	2.21.3	60	483	4818	Integration Framework
pulsar-client	2.8.0	277	1607	26076	Message Streaming
qpid-broker-j	7.1.0	1679	15738	189370	Messaging Middleware
qpid-jms	0.39.0	247	1290	17324	Messaging Middleware

**Table 7:** Projects in the *Web & App Frameworks* cluster (*n* = 8).

Project	Ver.	NOC	NOM	LOC	Domain
apache-flume	1.9.0	964	9643	109488	Event Collector
apache-roller	5.2.4	486	3772	59821	Blogging Platform
django	3.0.5	2274	17425	313806	Web Framework
grails	4.0.8	1456	12031	199539	Web Framework
playframework	2.8.2	1741	11897	236341	Web Framework
ruby-on-rails	6.0.3	1785	13642	284605	Web Framework
spring-boot	2.3.1	3011	19047	312951	Web Framework
vertx	3.9.1	1625	11203	203115	Reactive Toolkit

**Table 8:** Projects in the *Security & Identity* cluster (*n* = 7).

Project	Ver.	NOC	NOM	LOC	Domain
apache-accumulo	2.0.0	1598	13802	185963	Accumulo Store
apache-kylin	3.1.1	2161	18154	291114	OLAP Cube Engine
apache-ranger	2.1.0	1211	10801	132142	Security Service
keycloak	10.0.2	2184	19577	321613	Identity Mgt
openam	13.5.2	1376	11423	188567	Access Mgt
openssl	1.1.1g	2169	17102	237567	Crypto Library
vault	1.4.1	1086	9614	157420	Secrets Mgt

**Table 9:** Projects in the *Analytics & ML* cluster (*n* = 5).

Project	Ver.	NOC	NOM	LOC	Domain
apache-madlib	1.17	482	4271	62544	Analytics Library
apache-mxnet	1.7.0	3685	29105	495378	Deep Learning
apache-systemml	1.2.0	1873	16945	226862	Distributed ML
apache-tensorflow	2.2.0	6230	50211	749213	DL Framework
xgboost	1.1.0	919	6754	86603	Gradient Boosting

### Data Extraction

Once the projects were cloned, we used a tool called ANTLR4^30^ which is a powerful parser generator used to read, process, and analyze structured text or programming languages. It is commonly used to build language interpreters and compilers by converting source code into an Abstract Syntax Tree (AST) for easier analysis and transformation. In our case, the tool is used to parse the source code into an AST. From the AST, we extracted relevant information, such as method names, class names, and the bodies of methods and classes. While ANTLR4 offers the capability to extract additional details like code metrics, we limited our extraction to the necessary data at this phase. Numerical features were extracted in a subsequent step.

We then extracted code metrics from the textual code; these metrics are structural metrics of the code that can be helpful in training machine learning algorithms. The feature extraction process took 78 hours to complete. After extraction, the data was cleaned by removing comments, empty lines, and newline characters. Additionally, a Python script was used to calculate various code metrics for each code sample. The extracted metrics are listed in Table 10, and these metrics are^31^:

**Table 10:** Statistic for the columns.

Column	Min	Max	Mean
Logical Lines	1.00	69292.00	68.50
Distinct Operators	0.00	16.00	3.35
Distinct Operands	0.00	5112.00	44.77
Total Operators	0.00	65320.00	70.29
Total Operands	0.00	383408.00	368.96
Vocabulary	0.00	5123.00	48.12
Length	0.00	448728.00	439.25
Calculated Length	0.00	63016.22	305.30
Volume	0.00	5233964.63	3935.90
Difficulty	0.00	533.35	6.53
Effort	0.00	2790589493.80	1011685.89
Time Required (sec)	0.00	155032749.66	56204.77
Bugs	0.00	1744.65	1.31
Cyclomatic Complexity	1.00	49344.00	35.80

**Table 11:** Descriptive Statistics for the dataset.

Label	Count
Total Samples	107554
God Class	4333
Data Class	3284
Feature Envy	1996
Long Method	1566
Both God Class and Data Class	108
Both Feature Envy and Long Method	1153

Let:

- *η*_1_: **Number of unique operators**
- *η*_2_: **Number of unique operands**
- *N*_1_: **Total occurrences of operators**
- *N*_2_: **Total occurrences of operands**

### 1. Program Length

$${\bf{Program}}\,{\bf{Length}}=N={N}_{1}+{N}_{2}$$

**Definition:** The Program Length is the total number of occurrences of both operators and operands in the code. It provides a basic measure of the program’s size.

### 2. Program Vocabulary

$${\bf{Program}}\,{\bf{Vocabulary}}=\eta ={\eta }_{1}+{\eta }_{2}$$

**Definition:** The Program Vocabulary is the total number of unique operators and operands used in the code. It reflects the diversity of elements in the program.

### 3. Volume

$${\bf{Volume}}=V=N\times {\log }_{2}(\eta )$$

**Definition:** The Volume represents the size of the code in terms of bits required to represent it. It gives a measure of the cognitive load required to understand the program.

### 4. Difficulty

$${\bf{Difficulty}}=D=\frac{{\eta }_{1}}{2}\times \frac{{N}_{2}}{{\eta }_{2}}$$

**Definition:** The Difficulty metric reflects the complexity of the program based on the ratio of unique operators to operands. Higher values indicate more complex code that is harder to read and understand.

### 5. Effort

$${\bf{Effort}}=E=V\times D$$

**Definition:** The Effort is an estimate of the amount of mental effort required to develop or maintain the program. It combines both the volume and difficulty of the code.

### 6. Time Required to Program

$${\bf{Time}}=T=\frac{E}{18}$$

**Definition:** The Time Required to Program estimates the actual time (in seconds) needed to implement the code, based on the Effort metric.

### 7. Number of Delivered Bugs

$${\bf{Bugs}}=B=\frac{{E}^{\frac{2}{3}}}{3000}$$

**Definition:** The Number of Delivered Bugs estimates the number of potential errors in the code, based on the Effort metric. It is an approximate measure of software reliability.

### Data Annotation

To label the dataset as either smelly or non-smelly, we relied on three major works in this field.

- **Alkhaeir and Walter**^32^: used Eclipse plugin for static analysis to detect code smells. Their study involved 10 smells; however only two were used in our study, which are Feature Envy and Data Class as they are the only two that match our four included smells in this study.
- **Reis** *et al*.^33^: manually annotated code smells using 100 teams. They studied three smells, which are Feature Envy, God Class, and Long Method.
- **Sotto-Mayor** *et al*.^34^: used a tool called Organic (https://github.com/opus-research/ancient-organic) to detect 20 different code smells. However, in our study, we only selected four of them: Feature Envy, God Class, Long Method, and Data Class, since these four are the smells included in our study. Note that their selection process was on the file level; for smells at the class level, we selected the files that contain only one class. For the method level, they provided a percentage of the existence of the method level smell in the file; we only took files with 100% and 0% percentages and gave each method in that file a smelly label if the percentage is 100, otherwise not smelly.

After collecting the annotations from these studies, we cleaned the data, which contained the path to the code smell in each project and the corresponding smell type.

We applied three main data cleaning steps: **Unification,** **Removing Duplicates**, and **Removing the Missing Values**. As a result, we obtained four cleaned datasets, each corresponding to a specific smell type, with binary labels indicating whether a sample is smelly or non-smelly.

#### Unification

The data was unified by: 1) Removing the word “Apache” from project names, converting them to lowercase, and using hyphens to separate words in multi-word project names, 2) Simplifying project versions by omitting project names (e.g., converting camel1.2.4 to 1.2.4), 3) Modifying file paths by replacing slashes/backslashes with dots and removing file extensions (e.g., .java), 4) Converting class names to lowercase and extracting them when embedded in file paths, 5) Converting method names to lowercase and isolating the method name if the full header was provided, and 6) Standardizing labels by converting various forms of true/false to 1/0.

#### Removing Duplicates

In our case, duplicates can occur in two forms: 1) The same method or class annotated with the same smell, or 2) The same method or class annotated with different smells. We only considered the first type as duplicates, where the same sample has the same smell label. This is because a multi-label dataset will be constructed later to separate different smells. For instance, if Class A in Project X was identified twice with the “God Class” smell, only one instance was retained. However, if it was identified once with the “God Class” smell and once with the “Data Class” smell, both instances were kept.

#### Removing Missing Values

As part of the cleaning process, we removed all instances with missing information that relate the annotated smell to the source code.

The final output consists of a refined version of the labeled datasets, where each instance includes the following attributes: project name, project version, file path, class name, method name (for method-level smells), a “smelly” label (1 indicating smelly and 0 indicating non-smelly), and source (indicating the reference used in prior studies to determine the label). The dataset is now prepared to match labels with the extracted data from previous steps.

### Matching Labels

With both the annotated labels and the extracted dataset, we proceeded to match each code sample with its corresponding annotation. We used regular expressions (regex) to align the code samples with the collected labels. This process resulted in four sub-datasets, one for each smell type, containing the following attributes: project name, project version, file path, class name, method name (for method-level smells), “smelly” (with values 0 or 1 indicating the presence or absence of the smell), source (indicating the study used to determine the label), and source code (either the method code for method-level smells or the class code for class-level smells).

### Merging

We used Python to process and merge samples that shared the same project and code but had different smells from the four obtained binary smell datasets. The merging process involved introducing four columns, one for each smell, and marking each sample with 1 for its respective smell and 0 for the others. We then grouped the data by code and project information, summing the smell columns to create a one-hot encoded multi-smell dataset.

## Data Records

The constructed dataset comprises 107,554 samples collected from 103 open-source Java projects. This section provides a detailed description of both the dataset’s features and labels.

### Features

The dataset includes both numerical and textual features. The numerical features can be utilized to train traditional machine learning or deep learning models. In contrast, the textual features are suitable for fine-tuning language models for code smell detection tasks or prompting large language models.

#### Textual Feature

Each sample in the dataset includes a **Code** column, representing the code snippet exhibiting a code smell. The code can be a complete Java class or a method. To ensure consistency, the code samples have been cleaned by removing newline characters, empty lines, and comments.

#### Numerical Features

**Fig. 2:**
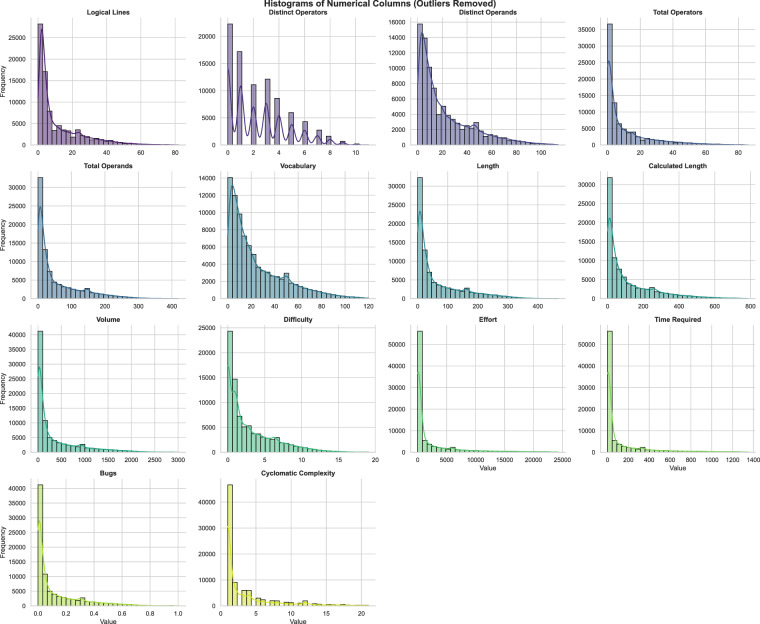
Histograms for the Numerical Data.

**Fig. 3:**
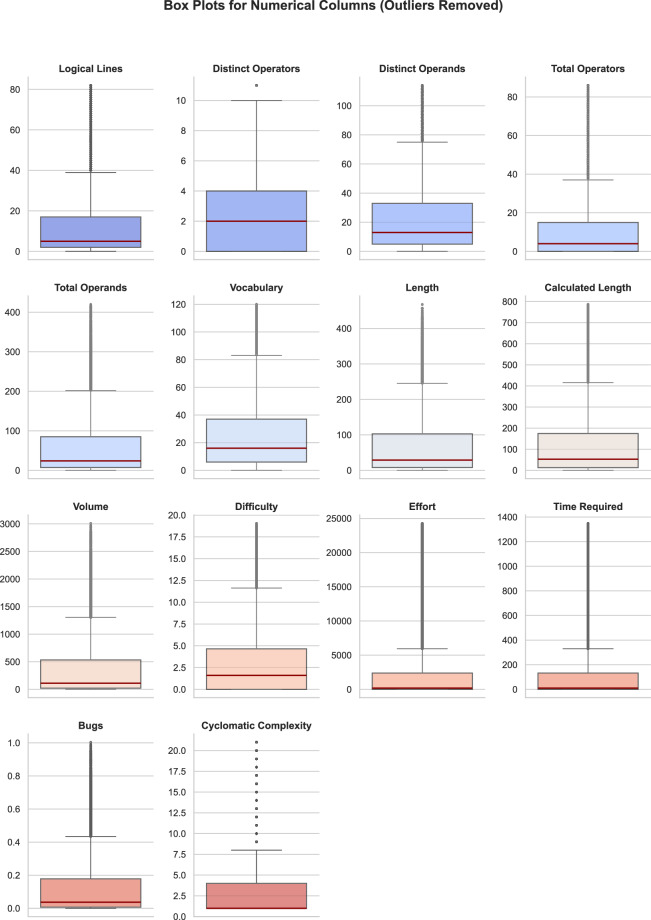
Box plots for the Numerical Data.

Each sample is accompanied by 14 numerical features, representing code metrics for the corresponding code snippet. These metrics include **Logical Lines, Distinct Operators, Distinct Operands, Total Operators, Total Operands, Vocabulary, Length, Calculated Length, Volume, Difficulty, Effort, Time Required, Bugs**, and **Cyclomatic Complexity**. The means and distributions of these features are presented in Table 10, while Figs. 2 and 3 provide a visual representation. Note that outliers were removed from the figures to enhance clarity; however, complete statistics, including outliers, are available in the table.

**Fig. 4:**
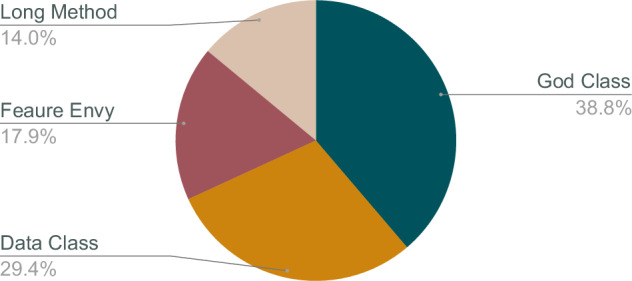
Smell Distribution in Smelly Samples.

**Fig. 5:**
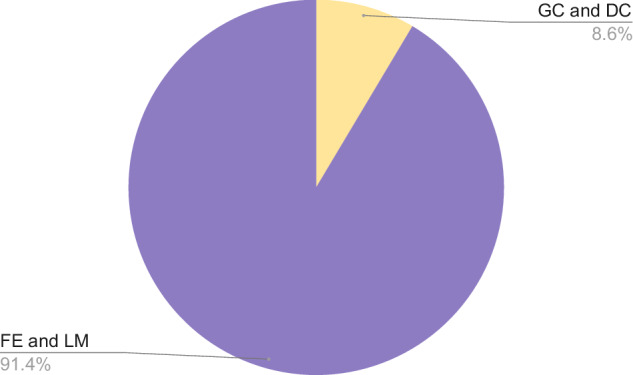
Multi Smells Distribution.

### Labels

The dataset is a multi-label dataset consisting of four distinct code smells, applicable at both the Class Level and Method Level. The code smells present in the dataset are God Class, Data Class, Long Method, and Feature Envy.

The labels in the dataset are represented using multi-hot encoding, similar to one-hot encoding. In multi-hot encoding, a vector is created with the same length as the number of labels, containing 1s and 0s to indicate the presence of specific labels. Unlike one-hot encoding, multiple positions in the vector can have a value of 1 for a single sample, indicating that the sample exhibits more than one code smell. The characteristics of each code smell, as well as their combinations, are detailed in Table 11. Fig. 4 visualizes the distribution of the smells in the dataset. Fig. 5 shows the distribution of the samples containing multiple smells.

#### Imbalanced Dataset

**Fig. 6:**
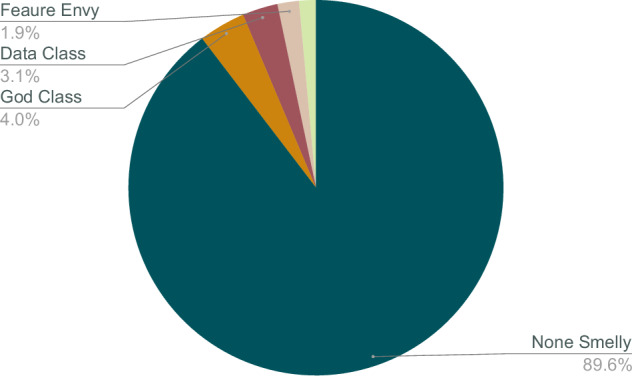
Smelly Vs None Smelly.

The dataset is imbalanced, as some code smells occur more frequently than others. To address this imbalance, techniques such as oversampling or undersampling can be employed during the training phase. Alternatively, a weighted loss function can be used to account for the imbalance and improve model performance. Fig. 6 shows that most of the samples in the dataset are not smelly.

## Technical Validation

Alazba *et al*.^13^ utilized this dataset to fine-tune a transformer-based code language model for code smell detection. They proposed a code language model that was pre-trained on 16 million lines of Java code from open-source projects. The model was subsequently fine-tuned using this dataset. For the fine-tuning process, the dataset was converted into a binary format, creating a subset for each code smell, where each subset indicated the presence or absence of a specific smell. Four models were trained in total, each corresponding to a different code smell.

### Preprocessing

The dataset was divided into training and testing sets with a 70:30 ratio. To address class imbalance, oversampling was applied using the SMOTE technique. For the textual code samples, tokenization was performed with a maximum sequence length of 256, making it suitable for input to the model.

### Training

The model was initially pretrained using a self-supervised learning approach, which does not require manually annotated labels. Subsequently, it was fine-tuned using supervised learning, where the model learns from labeled samples by predicting the corresponding labels and updating its weights based on a loss function. This loss is computed by comparing the predicted labels to the true labels. The model was trained for five epochs using the Adam optimizer with a learning rate of 0.001. The loss function employed was Binary Cross-Entropy Loss, which is commonly used for binary classification tasks.

1 $$L(y,\widehat{y})=-\frac{1}{N}\mathop{\sum }\limits_{i=1}^{N}\left[{y}_{i}\log ({\widehat{y}}_{i})+(1-{y}_{i})\log (1-{\widehat{y}}_{i})\right]$$

where:

- *y*_*i*_ is the true label (either 0 or 1).
- ${\widehat{y}}_{i}$ is the predicted probability that the label is 1.
- *N* is the total number of samples.

### Evaluation

Various evaluation metrics were employed to assess model performance on the test dataset, including Accuracy, Precision, Recall, F1 Score, Area Under the Curve (AUC), and Matthews Correlation Coefficient (MCC). These details of these metrics are as follows:

#### Evaluation Metrics

This section provides a formal definition of the evaluation metrics used to assess the performance of the models on the test dataset.

#### Accuracy

Accuracy measures the proportion of correctly predicted samples out of the total number of samples.

2 $$\,{\rm{Accuracy}}\,=\frac{TP+TN}{TP+TN+FP+FN}$$

where:

- *TP* = True Positives, the number of correctly predicted positive samples.
- *TN* = True Negatives, the number of correctly predicted negative samples.
- *FP* = False Positives, the number of incorrectly predicted positive samples.
- *FN* = False Negatives, the number of incorrectly predicted negative samples.

#### Precision

Precision, also known as Positive Predictive Value, measures the proportion of correctly predicted positive samples among all predicted positive samples.

3 $$\,{\rm{Precision}}\,=\frac{TP}{TP+FP}$$

It indicates the accuracy of the model in identifying true positives while minimizing false positives.

#### Recall

Recall, also known as Sensitivity or True Positive Rate, measures the proportion of correctly predicted positive samples among all actual positive samples.

4 $$\,{\rm{Recall}}\,=\frac{TP}{TP+FN}$$

It assesses the model’s ability to capture all true positive cases, reducing the number of false negatives.

#### F1 Score

The F1 Score is the harmonic mean of Precision and Recall. It balances both metrics, making it useful when the dataset is imbalanced.

5 $$\,{\rm{F1\; Score}}=2\cdot \frac{{\rm{Precision}}\cdot {\rm{Recall}}}{{\rm{Precision}}+{\rm{Recall}}}$$

It gives equal importance to Precision and Recall, providing a single score that considers both false positives and false negatives.

#### Area Under the Curve (AUC)

AUC represents the area under the Receiver Operating Characteristic (ROC) curve, which plots the True Positive Rate against the False Positive Rate at various thresholds. AUC ranges from 0 to 1, with 1 indicating perfect classification and 0.5 representing random performance.

6 $$\,{\rm{AUC}}={\int }_{0}^{1}{\rm{TPR}}\,(t)\,d{\rm{FPR}}\,(t)$$

where:

- TPR(*t*) is the True Positive Rate at the threshold *t*.
- FPR(*t*) is the False Positive Rate at the threshold *t*.

#### Matthews Correlation Coefficient (MCC)

MCC is a balanced measure for binary classification, taking into account True Positives, True Negatives, False Positives, and False Negatives. It ranges from  − 1 to 1, where 1 indicates perfect prediction, 0 indicates random prediction, and -1 indicates complete disagreement between prediction and actual labels.

7 $$\,{\rm{MCC}}\,=\frac{(TP\cdot TN)-(FP\cdot FN)}{\sqrt{(TP+FP)\cdot (TP+FN)\cdot (TN+FP)\cdot (TN+FN)}}$$

MCC is especially useful for imbalanced datasets, providing a comprehensive evaluation of model performance.

The results indicated that CoRT outperformed other models in detecting code smells, achieving an F1 Score of 95.89% for Data Class, 94.48% for God Class, 88.68% for Feature Envy, and 88.87% for Long Method. A detailed summary of performance metrics is presented in Table 12.

**Table 12:** CoRT Performance^16^.

Code Smell	Accuracy	Precision	Recall	F1	AUC	MCC
God Class	94.59	96.54	92.58	94.48	98.05	89.33
Data Class	95.93	96.82	95.12	95.89	98.33	91.99
Feature Envy	88.40	89.49	90.38	88.68	92.40	78.34
Long Method	87.95	87.10	92.11	88.87	90.21	77.16

## Threats to Validity

We have identified several potential threats that could affect the validity of our dataset construction. In this section, we outline these threats and describe the measures taken to mitigate their impact.

### Internal Validity

#### Labeling Bias

Internal threats are linked to the accuracy of the experimental results using this dataset, as similar code smells may have different names. To address this issue, we relied on the definitions provided by the authors of the original papers for dataset labeling. If a definition was not provided, we used the original definitions suggested by Fowler^3^.

### External Validity

These threats may affect the generalizability of our dataset. While we aimed to make our dataset as comprehensive as possible, there are some limitations:

#### Small Number of Code Smells

Our dataset includes only four code smells: God Class, Feature Envy, Long Method, and Data Class. Although this may appear to be a limited selection, these four smells were chosen due to their significant impact on code and software quality^35^. Furthermore, most of the selected smells are among the most used smells in code smell detection studies^36^

Future work could also extend this study to include more code smells.

#### Java Dataset

Another factor that could impact the generalizability of our dataset is its focus only on Java code. The decision to use Java was driven by its status as one of the languages with the most abundant open-source projects available^37^, making it a common choice in software engineering studies. Additionally, we ensured diversity by selecting 103 open-source projects from various domains and of varying sizes, aiming to make the dataset as representative as possible. Future work could also extend this study to include more programming languages to make it more general.

### Construct Validity

#### Text Prepossessing

One aspect that might impact the validity of our dataset is that we cleaned the code samples collected by removing new line characters and comments lines. We applied this processing as it can reduce the dataset size, removing unnecessary noise and size without affecting the training results, as shown by Alazba *et al*.^13^.

#### Dataset Validation

Another aspect is that we did not validate the labeling of the dataset; however, we used the labels from different published studies and relied on their validation as presented in those studies.

#### Metric Choice

An additional consideration is the limited selection of extracted code metrics. Nevertheless, we have focused on extracting the most widely used metrics for measuring software complexity^31^. Furthermore, we have chosen metrics that can be calculated on individual code snippets, whether classes or methods, as our dataset comprises both.

### Conclusion Validity

Threats to conclusion validity may affect the statistical significance of the constructed dataset. One potential threat is the limited number of source projects from which the dataset was collected. To mitigate this threat, we ensured diversity in the selected projects and collected data from over 107,000 instances. This large and varied sample size helps enhance the statistical significance and generalizability of our dataset.

## Data Availability

The code for constructing the dataset and extracting the code metrics can be found in the Figshare Repository^38^, and the code for analytics and dataset construction can be found in a public GitHub Repository (https://github.com/nawafalomari/code_smell_detection_dataset).
